# Research Audit on Clinical Utility of Dimensional Disruptive Mood and Behavior Psychopathologies in Child and Adolescent Psychiatry Practice

**DOI:** 10.3389/fpsyt.2022.742148

**Published:** 2022-04-06

**Authors:** Ji-Woo Suk, Katrina M. Poppert Cordts, William Garvey, Arica Lerdahl, Brigette Soltis-Vaughan, Alexandra Bohn, Ryan Edwards, Robert James Blair, Soonjo Hwang

**Affiliations:** ^1^Department of Psychiatry, University of Nebraska Medical Center, Omaha, NE, United States; ^2^Child and Adolescent Mental Health Centre, Mental Health Services, Capital Region of Denmark, Copenhagen, Denmark

**Keywords:** dimensional psychopathology, latent profile analysis (LPA), callous-unemotional trait, irritability, aggression

## Abstract

To investigate the utility of dimensional psychopathologies of disruptive mood and behavior disorders (DBDs) by applying latent profile analysis (LPA) for characterization of youth referred to the tertiary outpatient clinic of child and adolescent psychiatry clinic and pharmacological treatment choices. One hundred fifty-eight children and adolescents with significant DBDs symptoms participated. Core dimensional psychopathologies of DBDs (irritability, callous-unemotional trait, and reactive-proactive aggressive behavior), DSM diagnoses, prescribed medications, and behavioral and emotional problems (Child Behavior Checklist, CBCL) were measured at baseline (clinic intake) and at 3-month follow-up. Latent Profile Analysis (LPA) was applied to characterize the study population based on the levels and interrelations among the core dimensional DBDs psychopathologies. Following LPA, the differences in clinical and treatment features between the latent classes were analyzed. LPA revealed two latent classes based on severity of DBDs symptoms. Class 1 (the moderate group) was characterized by relatively low scores on all trans-diagnostic indicators, whereas class 2 (the severe/critical group) showed higher levels of the dimensional psychopathologies and the majority of CBCL subscales. In addition, the severe/critical group was more often prescribed antipsychotic medications, and also experienced more frequent medication changes (addition, increasing the dose, and trial of different medications). Our findings suggested that application of LPA to a cluster of dimensional DBDs psychopathologies may provide valuable characterization of the youths referred to a tertiary outpatient child and adolescent psychiatric clinic, and offer insight into the providers' decision making on psychotropic medications, by overall severity of these psychopathologies rather than by single categorical diagnosis or single externalizing psychopathology.

## Introduction

**Figure 1:**
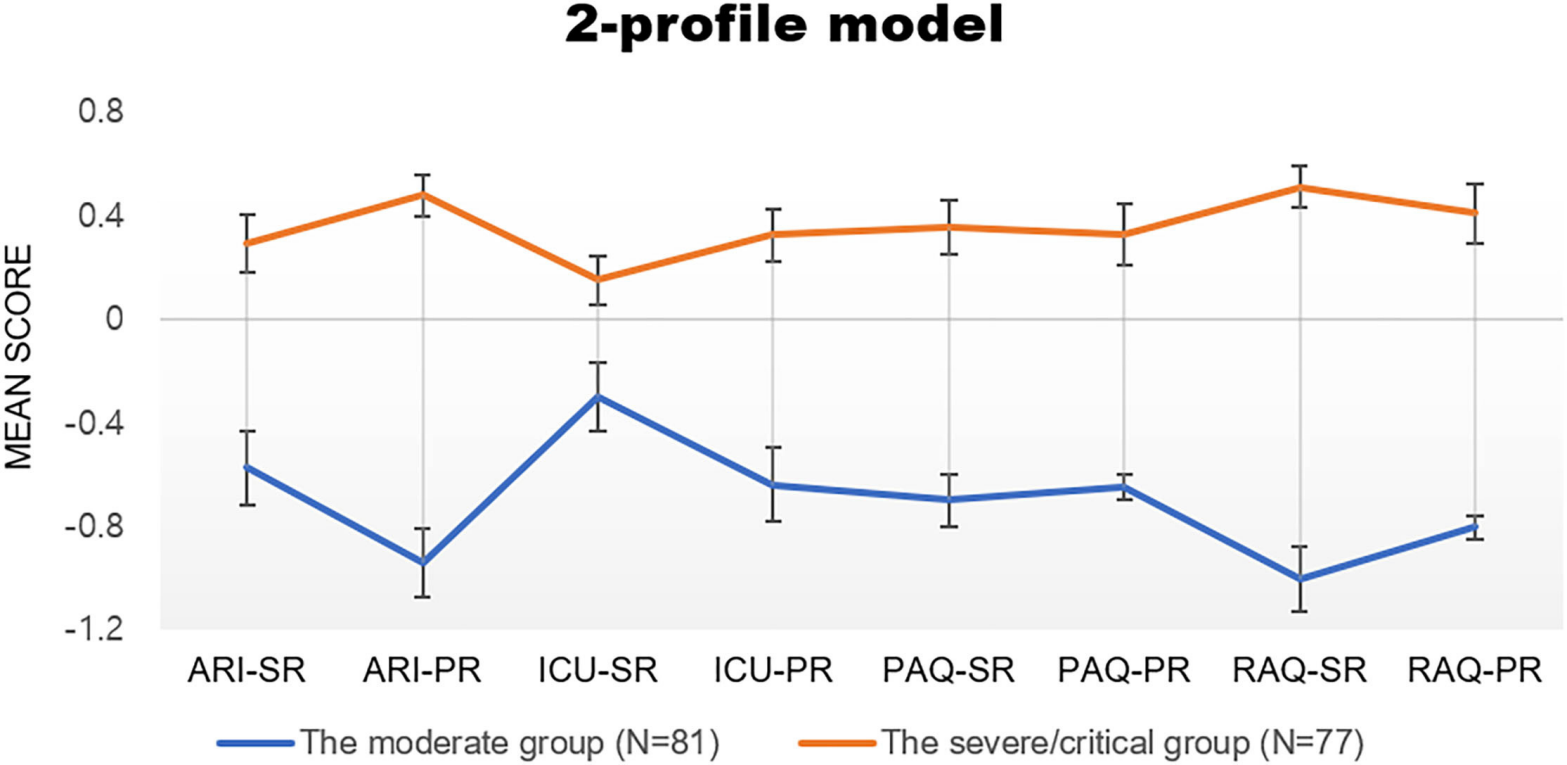
Mean score of class membership at baseline in each latent class. ARI-SR, ARI-PR, ICU-SR, ICU-PR, PAQ-SR, PAQ-PR, RAQ-SR, and RAQ-PR refer to indicators for LPA of aggression, irritability and callous-unemotional traits reported by youth and their caregivers. ARI, Affective Reactivity Index; AQ, Aggression Questionnaire ICU, Inventory of Callous Unemotional Traits; PR, parent-report; SR, self-report.

For the last three decades, there has been a steady and significant increase in the use of mental health care (1) and specifically psychotropic medications for pediatric patients (2). While there has been effort to determine efficacy of psychotropic medications for youths with mental health disorders (3), clinicians continue to face significant difficulties in providing optimal treatment. Indeed, there have been concerns raised about safe and appropriate use of psychotropic medication in pediatric patients (4, 5). One of the difficulties clinicians face is the complexity of patients they encounter where co-morbidities of categorical diagnoses is effectively the norm, specifically, see Figure 1 (6, 7). Theoretically, categorical diagnoses should provide guidance on medication choice [e.g., stimulant medications for Attention-Deficit/Hyperactivity Disorder (ADHD), serotonin selective reuptake inhibitor (SSRI) for depression, antipsychotic medication for schizophrenia, etc.,] (8). However, in reality, categorical diagnoses may have limitation on the identification of subgroups who might be more responsive to a treatment modality (9, 10). Moreover, heterogeneity in how the categorical disorders present at the case/individual level and the different co-morbidities shown by the majority of patients make this approach difficult to implement (8).

Our goal in this study was to assess how clinicians in a real-world setting, specifically a tertiary level outpatient clinic, prescribe psychotropic medications for pediatric mental health patients. In particular, we were interested in determining the extent to which co-occurring externalizing behavior/psychopathologies may influence treatment decision-making, because externalizing behavior/psychopathologies are the most common reason underpinning referral to pediatric mental health clinics (11). Given the very significant concern patients' families and their clinicians have regarding this symptom class, we suspect that co-occurring externalizing behavior might have a significant impact on treatment choice. Importantly, though, there are different forms of externalizing behavior/psychopathologies (12). Specifically, a distinction has been made between instrumental and reactive aggression (13). Instrumental aggression can occur with little or no overt provocation, in order to obtain a personal advantage, whereas reactive aggression implies a defensive response to perceived provocation (14, 15). Instrumental and reactive aggression have been particularly associated with callous-unemotional traits and irritability, respectively (16). Callous-unemotional traits include a relative lack of guilt/remorse and empathy for others (17). Irritability is characterized as an increased propensity to exhibit anger in response to frustration/non-reward relative to one's peers (18). Both callous-unemotional traits and irritability are seen in a variety of pediatric psychiatric diagnoses, including not only externalizing conditions [i.e., Oppositional Defiant Disorder (ODD), Conduct Disorder (CD) and ADHD] (19–21), but also internalizing conditions (particularly irritability) (21).

As such, we examined not only prescription practice as a function of mental health diagnoses but also as a function of externalizing psychopathologies. Given the associations between callous-unemotional (CU) traits, irritability, instrumental and reactive aggression, we applied a novel statistical method of latent profile analysis (LPA) which provides person-centered analysis on various variables to characterize a complex and heterogeneous population (22). We were particularly interested in the extent to which LPA might provide a valid characterization of the disruptive behavior disorder (DBDs) symptoms shown by the patients in this sample—and particularly the extent to which an LPA derived classification might be associated with psychotropic medication choices made by clinicians working with these patients.

To investigate the utility of dimensional psychopathologies, we recruited the youths with emotional and behavioral dysregulation. Emotional and behavioral dysregulation has been recognized as one of the most serious psychopathologies in children (23). It is one of the most common reasons children and adolescents present for mental health services (11, 24–26). The most prominent group of children whose main psychopathology is emotional and behavioral dysregulation are those with disruptive mood and behavior disorders (DBDs), including Attention Deficit Hyperactivity Disorder (ADHD) (20), Oppositional Defiant Disorder (ODD) (27), Conduct Disorder (CD) (28), and Disruptive Mood Dysregulation Disorder (DMDD) (29).

We made several predictions with respect to this research audit of clinical practice. First, we predicted that categorical diagnoses would predict medication choice; that is, (1) youths with ADHD would be more likely to be prescribed on stimulant medications and/or alpha-agonists than youths without ADHD diagnoses; (2) youths with diagnoses of depressive disorder and/or anxiety disorder would be more likely to be prescribed on SSRIs than youths without these diagnoses, and (3) youth with diagnoses of ODD and CD would be more likely to be prescribed on antipsychotic medication (for their aggression) than youths without these diagnoses. Second, and based on previous work (6, 30), we predicted that LPA would identify three categories of youth within the sample: (i) patients showing limited aggression, CU traits and irritability; (ii) patients showing significant aggression (instrumental and reactive) and elevated CU traits; and (iii) patients showing significant reactive aggression and irritability. Third, we predicted that the above LPA-identified categories would significantly predict providers' choice of psychiatric medications. Specifically, we predicted that patients showing significant aggression and elevated CU traits will be more likely to be prescribed on antipsychotic medication (for their aggression) than other psychiatric medications (31) while patients showing significant reactive aggression and irritability would be more likely to be prescribed on SSRIs than other psychiatric medications (32).

## Methods

### Procedures

Participating youths were recruited from the tertiary outpatient child and adolescent psychiatry clinic at a large academic medical center campus in the Midwest. Trained research assistants contacted the caregivers of eligible youths prior to their initial visit for psychiatric assessment/treatment, and provided information about the study. Inclusion and exclusion criteria for study participants were shown in the Supplementary Material. After obtaining written consent and assent, youths and their caregivers completed symptom profile measurements at baseline. Clinical characterization including psychiatric diagnosis was done through psychiatric interviewed by licensed and board-certified child and adolescent psychiatrists with participants and their parents, to adhere closely to common clinical practice. Participants' active psychiatric medications at the time of each study visit (baseline and 3-month follow-up) and their medications after each study visit (if their medications changed within 2 weeks of the visit by the providers) were recorded. The Institutional Review Board at the participating academic medical center approved the study procedures (UNMC IRB number: 586-18).

Diagnosis and psychiatric medication status were collected at each one of four data collection points [the initial visit (baseline), after the initial visit, the 3-months follow-up, and after the 3-months visit]. Symptom profiles and CBCL were collected at the initial visit and 3 months of follow-up. At the baseline, 158 youths provided information of sociodemographic characteristics and symptom profile measurements. Among them, 136 youths had identifiable information on diagnoses and medication at the initial visit. Twenty-two youths and their parents did not agree on providing information on their diagnoses or medication status. Of the four data collection points (before the initial visit, after the initial visit, before the 3 months visit, and after the 3 months visit), there were 83 youths who provided information of their diagnoses and medication status for all four data collection points, 15 for 3, 36 for 2, and 2 for only one data collection point, respectively. Among them, 15 patients didn't receive any medication (3 for all four data collection points, 3 for 3 and 9 for 2).

### Measures

#### Sociodemographic Information

Upon enrollment, caregivers and youths provided information about the participants' age, gender, and racial ethnic background. Using the electronic medical record, trained research assistants recorded mental health diagnoses at each study visit (baseline and 3-month follow-up).

#### Medication Recording

Participants' active psychiatric medications at the time of each study visit (baseline and 3-month follow-up) and their medications after each study visit (if their medications changed within 2 weeks of the visit by the providers) were recorded. Participants' medications were recorded from the electronic chart of the clinical visit documentation. Medications were also cross-checked in the medication list of the participant's medical record. Psychiatric medications included those prescribed or managed by psychiatrists or which were specifically prescribed for treating psychiatric symptoms.

#### Symptom Profile Measurements

Both the participants and their caregivers completed symptom profile measurements at the time of their visit to the clinic for psychiatric assessment/treatment. For youths requiring assistance, a trained research assistant facilitated completion by reading the questionnaires to the child.

The Reactive-Proactive Aggression Questionnaire (RAQ and PAQ, respectively) was administered as self- and parent-report to assess two types of aggressive behavior (33). Both the participant and caregiver completed the Affective Reactivity Index (ARI) to assess irritability and emotion dysregulation (34), and the Inventory of Callous Unemotional Traits (ICU) (17). Also, the Child Behavior Checklist for Ages 6–18 (CBCL/6-18) was administered to caregivers (35) at baseline, and 3-month follow-up visits. A detailed description of the measurements used is shown in the Supplementary Material.

### Statistical Analysis

#### Socio-Demographic and Clinical Characteristics

To characterize the study population, frequency distribution analysis was performed to identify the socio-demographic and clinical characteristics of all the participants.

#### Medication Choices by Categorical Diagnosis

To assess the medication choices (i.e., stimulant, SSRI, atypical antipsychotic, and alpha-adrenergic agonist) based on the categorical diagnosis, chi-square tests between the youths with and without a specific diagnosis were conducted, to examine the relationship between ADHD and stimulant/alpha-agonist medications, between Depressive Disorder/Anxiety Disorder and SSRI, and between ODD/CD and antipsychotic medications.

#### Identification and Description of the Latent Classes Derived From Symptom Profile

We conducted latent profile analysis (LPA) to identify distinct classes of individuals based on the unobserved latent profiles that generate patterns of responses on the three indicators (i.e., irritability, aggression, and callous-unemotional traits) (36). Latent profile models were tested using tidy LPA package version 0.2.4 (37) in R version 3.2.3 to find the optimal model. A detailed analysis method is provided in the Supplementary Material.

The optimal model has equal variances across classes and zero co-variances, as this is most in line with classical LPA and the default implementation in MPlus (38).

We standardized each variable using z-scores for clarity of interpretation. Then, LPA was conducted to determine latent, or unobserved, profile membership based on an individual's observed responses on each of the eight indicators including self- and parent- report of ARI, self- and parent- report of ICU, self- and parent-report of proactive AQs, and self- and parent-report of reactive AQs.

Generally, the number of latent profiles is determined through exploratory procedures by evaluating a number of different candidate models based on classification quality, parsimony indexes, and relative goodness of fit of two competing statistical models for the optimal model selection. For the study, a number of different candidate models were evaluated based on classification quality by entropy, parsimony indexes including Akaike Information Criterion (AIC) (39), Bayesian Information Criterion (BIC) (40), and Integrated Complete Likelihood (ICL) (41), and relative goodness of fit of two competing statistical models using Bootstrap Likelihood Ratio Test (BLRT) (42) for the optimal model selection.

#### The Differences of Symptom Profile and Socio-Demographic/Clinical Characteristics Between the Latent Classes

After the latent profile model was identified, differences in the symptom profiles, socio-demographic and diagnosis, and CBCL subscales were analyzed between the latent groups with chi-square test, *t*-test, and Mann-Whitney *U-*test.

Also, a series of repeated measures analysis of variance were conducted to assess whether there were group difference (between latent groups) and two assessment points (baseline and 3-month follow-up) on the eight symptom profile measurements.

#### Medication Choices by LPA Identified Groups

To assess the medication choices (i.e., stimulant, SSRI, atypical antipsychotic, and alpha-adrenergic agonist) and medication changes for 3 months based on the LPA identified groups, two sample *t*-test and chi-square tests between the latent classes were conducted.

#### Additional Analysis 1: Correlation of the Dimensional Psychopathologies

Because the LAP analysis did not divide the groups as we hypothesized (high irritability-low callous-unemotional trait-high reactive aggression vs. high callous-unemotional trait-high proactive/reactive aggression), we conducted additional analyses of the correlation of these psychopathologies in the two groups, to determine the relations between different types of aggression (reactive vs. proactive), irritability, and callous-unemotional trait.

#### Additional Analysis 2: LPA Analysis With Externalizing and Internalizing Psychopathologies

Due to the significant number of the participants who presented with internalizing diagnoses as their primary diagnoses (see Table 1), we conducted additional LPA analyses including CBCL internalizing psychopathologies in addition to the three disruptive mood and behavior psychopathologies. In the analyses including (1) overall internalizing problems, (2) withdrawn-depression, depressive problems, and anxiety problems, and (3) anxious-depressed score, withdrawn-depressed score, depressed problems, and anxiety problems.

**Table 1:** Descriptive statistics on the socio-demographic characteristics of the subjects (*N* = 158).

**Variable**	***N* (%)**	**%**
**Age (yr)**		
Under 6	4	2.53
Older than 6~under 11	36	22.78
Older than 11~under 14	35	22.15
Older than 14~under 18	76	48.10
Older than 18	7	4.43
**Gender**		
Boy	81	51.27
Girl	75	47.47
NM	2	1.27
**Race** ^**a**^		
White	121	76.58
African American	12	7.60
Mixed	11	6.96
Others	14	8.86
**Ethnicity**		
Not hispanic	141	89.24
Hispanic	17	10.76
**Diagnosis**^**b**^ **(*****N*** **= 136)**		
ADHD	65	47.79
Anxiety	60	44.12
Depressive	63	46.32
CD/ODD	16	11.76
Trauma and stress-related	15	11.03
Autism spectrum	13	9.56
Communication	10	7.35
Elimination	5	3.67
Specific learning	5	3.67
Others	32	23.53
**Medication**^**c**^ **(*****N*** **= 136)**		
Stimulant	40	29.41
SSRI	38	27.94
Atypical Antipsychotics	24	17.65
Alpha adrenergic agonist	22	16.18
Anticonvulsant	10	7.35
Supplements	16	11.76
Others	11	8.09

a *Others in race include Asian, Latinx, Native American; Mixed in Race includes Caucasian/Black, Caucasian/Other, Caucasian/Asian, Caucasian/Latinx, Latinx/Other etc*.

b *Others in mental disorder include Feeding and Eating, Conversion, Personality, Elimination*.

*Excoriation, Intellectual Disability Obesity, Obsessive Compulsive, Schizophrenia and Psychotic, Sleep-Wake, Substance Use, and Tic*.

c *Supplements in Medication include Acetylcysteine and Melatonin; Others include Non-Stimulant ADHD, Antianxiety, Benzodiazepine, Antihistamine, Non-SSRI Antidepressant and Typical antipsychotics*.

*ADHD, Attention Deficit Hyperactivity Disorder; CD, Conduct Disorder; NM, Not Mentioned; ODD, Oppositional Defiant Disorder*.

## Results

### Socio-Demographic/Clinical Characteristics and Medication Change in All Participants

A total of 158 youths (51.27% male) who were referred to the outpatient child and adolescent psychiatry clinic participated in this study. Most of the participants were White (76.58%) and non-Hispanic (89.24%). Youths were predominantly junior high school students (48.10%) and an average of 13.50 years of age [Standard Deviation (SD) = 3.32]. The prevalence of mental disorders (*N* = 136) was as follows: ADHD (49.79%), depressive disorders (46.32%), anxiety disorders (44.12%) and CD/ODD (11.76%); see Table 1.

Prescribed medications from the youths and parents who provided information on this at the initial visit (N=136) were as follows: stimulant (29.41%), SSRI (27.94%), atypical anti-psychotics (17.65%), and alpha-adrenergic agonists (16.18%). Thirty-two youths (23.53%) took more than one medication among stimulant, SSRI, atypical anti-psychotics, and alpha-adrenergic agonists; see Table 1.

### Medication Choices by Categorical Diagnoses

Table 2 shows cross-tabulation of medication choices by categorical diagnoses. As predicted, participants with ADHD had a significantly higher number of stimulant (60%) and alpha-adrenergic agonist (29.24%) medications prescribed for them than youths without ADHD diagnosis (Stimulant: 1.4%, *X*^2^ = 56.11, *p* < 0.001 and alpha-adrenergic agonists: 4.23%, *X*^2^ = 15.65, *p* < 0.001). SSRIs were significantly more often prescribed for depressive youths (44.44%) than youths without depression (13.70%, *X*^2^ = 15.88, *p* < 0.001) but not for youths with anxiety diagnoses (anxiety group 33.33%, non-anxiety group 23.68%, *X*^2^ = 2.55, *p* = 0.11). Participants with CD/ODD were not significantly more likely to be prescribed atypical antipsychotics than youths without these diagnoses (CD/ODD group 31.25%, non-CD/ODD group 15.83%, *X*^2^ = 2.30, *p* = 0.13). However, youths with CD/ODD were significantly more likely to have alpha-adrenergic agonist (43.75%) prescriptions than youths without CD/ODD (12.5%, *X*^2^ = 10.17, *p* = 0.001).

**Table 2:** Cross tabulation of medication choices and categorical diagnoses (*N* = 136).

**Diagnoses**	**Medication**	**Prescription in other diagnoses, *n* (%)**	**Prescription in this diagnosis, *n* (%)**	**Value** **(χ*^**2**^*)**
ADHD (*n* = 65)	Stimulant	1 (1.40)	39 (60)	56.11^***^
	AAA	3 (4.23)	19 (29.24)	15.65^***^
Depressive (*n* = 63)	SSRI	10 (13.70)	28 (44.44)	15.88^***^
Anxiety (*n* = 60)	SSRI	18 (23.68)	20 (33.33)	2.55
CD/ODD (*n* = 16)	AA	19 (15.83)	5 (31.25)	2.30
	AAA	15 (12.5)	7 (43.75)	10.17^**^

*AA, Atypical Antipsychotics; AAA, Alpha Adrenergic Agonist; ADHD, Attention Deficit Hyperactivity Disorder; CD, Conduct Disorder; ODD, Oppositional Defiant Disorder; SSRI, Selective Serotonin Reuptake Inhibitor*.

** *p < 0.01, and*

*** *p < 0.001 for group comparisons*.

### Identification and Description of the Latent Classes Derived From Symptom Profile

We predicted that LPA would identify three categories of youth within the sample: (i) patients showing limited aggression, CU traits and irritability; (ii) patients showing significant aggression (instrumental and reactive) and elevated CU traits; and (iii) patients showing significant reactive aggression and irritability.

Table S1 shows the LPA fit indices is identified by increasing the number of latent classes sequentially to determine the optimal latent model. The two-class solution was selected, by considering the ICL value, the posterior probability and the proportions of members in each class. A description of the model selection is presented in the Supplementary Material.

In a model consisting of two latent classes selected in this study, each individual was assigned to classes based on their model posterior probabilities and adjustments for classification error when estimating the class-specific distributions (43). As a result, 81 (51.27%) of the total youths were allocated for latent class 1 and 77 (48.73%) for latent class 2. The symptom profile characteristics of these classes were examined and class labels were assigned based on these characteristics (Figure 1). Both classes had clinically significant levels of all symptoms, but class 1 comprised significantly lower mean levels of all indicators than class 2 (*p* < 0.001). Thus, class 1 was labeled the moderate group and class 2 was labeled the severe/critical group; see Figure 1.

### The Differences of Symptom Profile and Socio-Demographic/Clinical Characteristics Between the Latent Classes

The severe/critical group showed higher scores in all symptom profiles than the moderate group including irritability, callous-unemotional trait, and aggressive behavior (*p* < 0.001); see Table 3. Symptom profile changes in follow-up is shown in the Supplementary Material and Tables S2, S3.

**Table 3:** Descriptive statistics on the indicator variables in each group (*N* = 158).

**Variables**	**The moderate group (*N* = 81)**	**The severe/critical group (*N* = 77)**	**Value (*t* or *U*)**
Self-reported ARI	3.49 (±2.71)	6.82 (±2.83)	1255.5^***^
Parent-reported ARI	3.48 (±2.85)	7.01 (±2.88)	1232.0^***^
Self-reported ICU	21.79 (±7.63)	27.48 (±8.63)	4.40^***^
Parent-reported ICU	23.83 (±11.45)	32.97 (±10.38)	5.25^***^
Self-reported PAQ	6.90 (±3.17)	13.14 (±4.15)	739^***^
Parent-reported PAQ	7.73 (±4.11)	15.29 (±3.77)	590.5^***^
Self-reported RAQ	0.60 (±0.77)	4.32 (±3.46)	677^***^
Parent-reported RAQ	1.10 (±1.20)	6.41 (±4.07)	578.5^***^

*** *p < 0.01 for group comparisons*.

*ARI, Affective Reactivity Index; AQ, Aggression Questionnaire; ICU, Inventory of Callous Unemotional Traits*.

*The variables including ICU self-report and ICU parent-report are analyzed using two sample t test. The other variables are non-normal distributed and the Mann-Whitney U-test was used to compare between groups for the variables*.

As for the socio-demographic characteristics, the severe/critical group were more likely to be male (*X*^2^ =8.54, *p* = 0.004) compared to the moderate group. There was no significant age and racial/ethnic differences between groups. The severe/critical group was more likely to have a diagnosis of ADHD (*X*^2^ =5.63, *p* = 0.02) and CD/ODD (*X*^2^ = 6.00, *p* = 0.01) than the moderate group; see Table 4.

**Table 4:** Differences in socio-demographic and diagnosis between two Latent Classes (*N* = 158).

**Variables**	**The moderate group (*N* = 81)**	**The severe/critical group (*N* = 77)**	**Value (*U* or *X*^2^)**
**Age (yr)** ^**a**^	14.4 (12.1;16.3)	13.1 (10.1;16.5)	2626.5
**Gender**			
Boy	33(41.8%)	48(62.3%)	8.54^*^
Girl	46(58.2%)	29(37.7%)	
**Race** ^**b**^			
White	64 (79.0%)	57 (74.0%)	8.32
African American/Mixed	10 (1.2%)	13 (2.6%)	
Other	7 (3.7%)	7 (5.2%)	
**Ethnicity**			
Not hispanic	71 (87.7%)	70(90.9%)	0.44
Hispanic	10 (12.3%)	7 (9.1%)	
**Diagnosis**^**c**^ **(*****N*** **= 136)**	73	63	
ADHD	28 (38.36%)	37 (58.73%)	5.63^*^
Anxiety	36 (49.32%)	24 (38.10%)	1.73
Depressive	39 (53.42%)	24 (38.10%)	3.20
CD/ODD	4 (5.48%)	12 (19.05%)	6.00^*^
TSR	10 (13.70%)	5 (7.94%)	1.14
Autism spectrum	5 (6.85%)	8 (12.70%)	1.34
Communication	3 (4.11%)	7 (11.11%)	2.43
Elimination	2 (2.74%)	3 (4.76%)	0.39
SR	4 (5.48%)	1 (1.59%)	1.45

* *p < 0.05 for group comparisons*.

a *The age variable was non-normal distributed and the Mann-Whitney U test was used to compare between groups for the variables*.

b *Other in race includes Asian, Latinx, Native American; Mixed in Race includes Caucasian/Black, Caucasian/Other, Caucasian/Asian, Caucasian/Latinx, Latinx/Other etc*.

c *The number of diagnosis and prescription reports: The moderate group, N = 73; The severe/critical group, N = 63*.

*ADHD, Attention Deficit Hyperactivity Disorder; AS, Autism Spectrum; CD, Conduct Disorder; ODD, Oppositional Defiant Disorder; SR, Specific Learning; TSR, Trauma and Stress-Related disorder*.

In addition, the severe/critical group also showed significantly higher scores on symptom profiles measured by CBCL; see Figure S1 and Table 5.

**Table 5:** Differences in CBCL scores between two Latent Classes (*N* = 158).

**Variables**	**Categories**	**The moderate group (*N* = 81)**	**The severe/critical group (*N* = 77)**	**Value** **(*U* or *t*)**
Competence scale	Activities^a^	40.0 (34.5; 45.0)	42.0 (33.0; 49.0)	2630
	Social^a^	39.0 (30.0; 48.0)	32.5 (28.0; 45.0)	1954.5^**^
	School^a^	43.0 (37.0; 54.5)	38.0 (30.0; 46.0)	1948^***^
	Total competence^a^	36.0 (30.0; 46.0)	31.5 (26.0; 39.0)	2139.5^*^
Syndrome scale	Anxious depressed^a^	67.0 (57.0; 71.0)	68.0 (58.0; 75.0)	2461.5
	Withdrawn depressed^a^	66.0 (59.0; 71.5)	66.0 (60.0; 70.0)	2746
	Somatic complaints^a^	57.0 (53.0; 65.0)	62.0 (54.0; 72.0)	2215.5^*^
	Social problems^a^	58.0 (51.0; 67.0)	68.0 (61.0; 75.0)	1415.5^***^
	Thought problems^a^	64.0 (57.0; 69.0)	69.0 (62.5; 74.5)	2009.5^**^
	Attention problems^a^	61.0 (52.0; 69.0)	67.0 (64.0; 77.0)	1666^***^
	Rule breaking behavior^a^	51.0 (50.0; 58.0)	64.0 (60.0; 70.5)	1023.5^***^
	Aggressive behavior^a^	54.0 (50.0; 61.5)	68.0 (63.0; 77.0)	831.5^***^
Internalizing, externalizing, total problems	Internalizing problems^a^	67.0 (60.0; 71.0)	69.0 (61.0; 74.5)	2384
	Externalizing problems	53.8 ± 10.4	68.0 ± 7.9	9.31^***^
	Total problems^a^	63.0 (54.0; 68.0)	71.0 (64.5; 75.0)	1270^***^
DSM-oriented scale	Depressive problems	66.4 ± 9.1	70.5 ± 9.9	2.65^**^
	Anxiety problems^a^	63.0 (55.0; 73.0)	68.0 (57.5; 79.0)	2321
	Somatic problems^a^	54.0 (50.0; 62.0)	61.0 (50.0; 68.0)	2063^**^
	Attention deficit hyperactivity problems^a^	59.0 (51.5; 67.0)	68.0 (64.5; 72.0)	1495.5^***^
	Oppositional defiant problems^a^	55.0 (51.0; 62.0)	67.0 (62.0; 73.0)	1084^***^
	Conduct problems^a^	51.0 (50.0; 59.5)	67.0 (60.0; 73.5)	898^***^
2007 scale	Sluggish cognitive tempo^a^	61.0 (50.0; 66.0)	63.0 (55.0; 70.0)	2315.5
	Obsessive compulsive problems^a^	62.0 (55.0; 69.0)	64.0 (59.0; 71.5)	2498.5
	Stress problems^a^	64.0 (58.0; 71.0)	70.0 (64.0; 76.0)	1811^***^

* *P < 0.05*.

** *P < 0.01*.

*** *P < 0.001*.

*CBCL, Child Behavior Checklist*.

a *The variables are non-normal distributed and are described as median and interquartile range. The Mann-Whitney U-test was used to compare between groups for the variables*.

### Medication Choices by LPA Identified Groups

We examined the use of antipsychotic and SSRI medications in the groups identified by LPA. The severe/critical group (26.98%) were significantly more likely to be prescribed atypical antipsychotic medication compared to the moderate group (9.59%, *X*^2^ = 7.04, *p* = 0.01). In addition, the severe/critical group was more prescribed on alpha-adrenergic agonist compared to the moderate group (23.81% vs. 9.59%, *X*^2^ = 5.04, *p* = 0.02) (Table 6). However, there is no differences in the SSRI prescription between the severe/critical (22.22%) and the moderate group (32.88%, *X*^2^ = 1.91, *p* = 0.17).

**Table 6:** Medication choices and changes for three months in each latent group.

**Variables**	**Categories**	**The moderate group**	**The severe/critical group**	**Value (*t* or *X*^2^)**
**Prescription** ^**a**^	Stimulant	19 (26.03%)	21 (33.33%)	0.87
	AA	7 (9.59%)	17 (26.98%)	7.04^**^
	AAA	7 (9.59%)	15 (23.81%)	5.04^*^
	Anticonvulsant	3 (4.10%)	7 (11.11%)	2.43
	SSRI	24 (32.88%)	14 (22.22%)	1.91
	Supplements	9 (12.32%)	7 (11.11%)	0.05
**The difference of the mean of medication changes rate between groups** ^**b**^	-	0.49 ± 0.36	0.67 ± 0.36	2.89^**^
**Dose change in each drug after initial visit** ^**b**^	Increase	28 (38.88%)	34 (58.83%)	3.41
	No change	18 (25%)	9 (14.51%)	2.28
	Decrease	10 (13.89%)	9 (14.51%)	0.01
**Use of additional drugs** ^**b**^ ^,^ ^**c**^	-	29 (40.28%)	38 (61.29%)	5.88^*^

a *The number of diagnosis and prescription reports: The moderate group, N = 73; The severe/critical group, N = 63*.

b *The number of diagnosis and prescription reports: The moderate group, N = 72; The severe/critical group, N = 62*.

c *The type of additional drugs: moderate group, 51.72% SSRI, 17.24% Stimulant, 3.44% Atypical antipsychotics, 10.34% Alpha adrenergic agonist; severe/critical group, 34.21% SSRI, 36.84% Stimulant, 10.52% Atypical antipsychotics 26.32% Alpha adrenergic agonist*.

* *p < 0.05 and*

** *p < 0.01 for group comparisons*.

In addition, we found that the rate of medication changes in the severe/critical group (0.67) was higher than that of the moderate group (0.49) (*t* = 2.89, *p* = 0.004) during the 3-months study period. The severe/critical group was more likely to have additional medications, such as stimulants (36.84%) and atypical antipsychotics (10.52%), but also SSRI (34.21%) or alpha-adrenergic agonists (26.32%) (*X*^2^ = 5.88, *p* = 0.02) (Table 6).

### Additional Analysis 1: Correlation of the Dimensional Psychopathologies

In the study group as a whole, there were positive associations between all four symptoms (ARI, ICU, RAQ, and PAQ; the correlation coefficient ranges from 0.37 to 0.79, *p* < 0.01). In the moderate group, only ICU and RAQ did not show positive correlation. In the severe/critical group, ICU-ARI and ICU-RAQ did not show positive correlations (Tables S4–S6). When the degrees of correlation were compared, ARI had significantly stronger correlation with RAQ than ICU (Z-score = 6.88, 3.35, and 4.26 within all participant, the moderate group and the severe/critical group, respectively, *p* < 0.001) or PAQ (Z-score = 5.31, 2.37, and 3.68, within all participant, the moderate group and the severe/critical group, respectively, *p* < 0.001 or *p* < 0.01); see Tables S7–S10.

### Additional Analysis 2: LPA Analysis With Externalizing and Internalizing Psychopathologies

The results of the LPA, including other indicators relevant to internalizing symptoms (i.e., internalizing problems, withdrawn-depressed score, depressive problems, anxiety problems, or anxious-depressed score), can be found in Tables S11–S13. Even though other indicators were included for LPA, all yielded repetition of two-class solution (severe/critically ill vs. moderately ill) as the best fitting models; see Tables S11–S13 and Figures S2–S4. Since the entropy values indicate a similar fit for all class solutions, they did not help much in choosing among the models. AIC values indicated a similar fit for the two-, and three-class solutions. However, BIC- and ICL-values were inspected, with these values indicating the two-class solution to perform best. Also, the BLRT *p*-value was significant for only one-class model, which was the comparison result between the two-class and the one-class models, and other BLRTs failed to reach significance comparing the other classes models (at α = 0.05). Taken together, the two-class model seemed the most relevant to inspect.

## Discussion

In this study, we aim to identify the utility of dimensional disruptive behavior and mood psychopathologies in characterizing pediatric patients who were referred for psychiatric assessment/treatment, and its utility in provider's decision making, especially choice of psychotropic medications. To achieve this, we applied LPA to classify children and adolescents by their trans-diagnostic disruptive mood and behavior psychopathologies (i.e., irritability, callous-unemotional trait and reactive/proactive aggression) and assessed whether the subclasses differed significantly in clinical characterization as well as provider's medication treatment choices.

There are three main findings: (1) As we predicted, categorical psychiatric diagnoses of ADHD and depressive disorders were significantly related to being prescribed on stimulant/alpha-agonist medications and SSRIs, respectively. However, there was no further relation between categorical diagnoses and medication choices, especially including prescription of antipsychotic medications with or without diagnoses of ODD/CD. (2) There were two latent groups (critical/severe and moderate) explicitly distinguished by the severity of their irritability, callous-unemotional traits, and aggressive behavior, but not by form of symptom as we had expected. Our hypothesis of distinguishing youths as high irritability/low callous-unemotional trait/high reactive aggression vs. high callous-unemotional trait/high proactive/reactive aggression was not affirmed. (3) Lastly, the latent groups were associated with the medication choices made by the providers; the severe/critical group showed significantly higher rates of prescriptions for atypical antipsychotics and alpha-adrenergic agonists. Also, the severe/critical group showed significantly higher rates of medication changes and being prescribed additional psychiatric medications.

The first hypotheses on the role of categorical diagnoses in medication decision was partially confirmed. Stimulant and alpha-adrenergic agonist have long been used in youths with ADHD (44) and antidepressants, particularly the SSRIs, for youths with depression and anxiety disorders (45, 46). However, diagnosis of anxiety disorders was not related to prescription of SSRIs, which might be due to the fact that the non-anxiety group included significant numbers of depressive youths without comorbid anxiety (*n* = 30). In addition, we failed to see any relation between prescription of atypical antipsychotic and diagnoses of CD/ ODD, which has been a common practice for their aggressive/violent behavior (47). This might be a type II error, due to the small sample size in this category (*n* = 16).

Secondly, the results of LPA demonstrated that, contrary to our hypothesis, latent groups were distinguished by severity of overall psychopathologies, rather than by pattern of the externalizing psychopathologies. The subgroups did not reveal the potential dichotomy of high irritability/low callous-unemotional trait/high reactive aggression vs. high callous-unemotional trait/high proactive aggresion as we expected (6, 30). However, it should be also pointed out that the correlation analyses revealed that there was significantly stronger correlation between irritability and reactive aggression, than irritability and proactive aggresison or callous-unemotional trait, which partially affirmed our hypothesis 1 (see Tables S4–S6).

The subgroups (moderate vs. serious/critical) identified by LPA provided intriguing clinical points. Class 1 (the moderate group) was characterized by relatively low scores on all trans-diagnostic indicators and the youths in this group were more likely to be girls, and to be rated higher on the social competence domain, school competence domain, total competence domain among the CBCL subscales. In contrast, those in class 2 (the severe/critical group) showed higher scores on all trans-diagnostic indicators, and were more likely to be boys, to have diagnoses of ADHD and CD. Also, they showed higher scores on almost every subscale of CBCL. This finding might suggest that, the clusters of the external psychopathologies together (irritability, callous-unemotional trait, and reactive/proactive aggression) may provide better explanation of a couple of aspects of the patients referred to the tertiary child and adolescent psychiatry clinic including: (1) Overall severity of the patients driven by all these external psychopathologies and (2) Medication choices (especially atypical antipsychotics as well as frequency of medication changes/addition of psychiatric medications).

Previous studies based on variable-centered approaches including factor analysis have suggested that childhood psychiatric disorders can be divided into internalizing and externalizing categories (48). However, in this sample, the severe/critical group scored significantly higher in all the symptom profiles in the CBCL including internalizing psychopathology (depressive problems, anxious depressive symptoms, and anxiety problems; see Table 5) compared to the moderate group. This indicates that at least for patient clusters differentiated by external psychopathologies (irritability, callous-unemotional trait, and aggressive behavior), internalizing behavior problem were also higher in the group with severe externalizing problems. This may be unique to the patient group referred to the tertiary child and adolescent psychiatry clinic, and future study is warranted by including larger group of children and adolescents with internalizing problems. Rather, the overall severity of disruptive behavior and irritability can better characterize the patient popupation. Recently, the National Institute of Mental Health Research Domain Criteria initiative conducted an LPA using transdiagnostic symptom profiles, including irritability, anxiety, depressive, and ADHD symptoms, on youths with disruptive mood dysregulation disorder, anxiety disorder, ADHD, bipolar spectrum disorder to derive a class or pattern of psychiatric symptoms for youths that cross conventional nosologic boundaries (49). In line with our finding, they observed latent groups showing high or low levels of overall symptomaology but not “pure” classes exhibiting elevations in only one, or in a cluster of, symptom dimension(s). These findings highlight the need to consider the level of symptoms within individuals, rather than focusing solely on the presence of disorder specific symptoms for decision making of clinical characterization and treatement. Future studies are warranted to validate the utility of this approach for a large youth population with mental health issues in general.

We were able to find that the groups identified by the LPA application on the dimensional psychopathologies provided insightful explanation on the clinical practice of psychiatric medications for youths with disruptive mood and behavior symptoms.

First, SSRIs were more prescribed in the moderate group although this was not statistically significant (32.88 vs. 22.22% in the severe/critical group). However, at the same time it is noteworthy that some of the internalizing psychopathologies showed higher scores in the severe/critical group (depressive problems with statistical significance, and internalizing problems, anxiety problems, as well as anxious depressed; see Table 5). It is possible that the providers may tend to focus on depressive/anxiety symptoms when there is lesser degree of disruptive mood/behavior symptoms (although objective scores indicate that the severe/critical group also scores high or even higher on anxiety/depressive symptoms) since disruptive mood/behavior symptoms may tend to display themselves more explicitly during the clinical visit for assessment.

Second, alpha adrenergic agonists and antipsychotics have shown the effectiveness on treatment of comorbid symptoms of ADHD (38, 39), especially aggression, irritability, and low frustration tolerance (40, 41). Thus, it seems clinically justifiable that the providers at the tertiary level clinic choose alpha adrenergic agonists when there were severe symptom presentations, which were mainly driven by disruptive mood and disruptive psychopathologies, even in youths with primary diagnoses of Anxiety and Depressive Disorders (see Table 6). However, overall effectiveness of alpha-adrenergic agent in reducing the severity of symptom presentations driven by disruptive mood and behavior psychopathologies remain to be determined by further studies.

In addition, the providers at this clinic tended to choose antipsychotic medications when youths present more severe/critical symptom presentations overall. Antipsychotic medications have been used for severe aggression especially in the diagnoses of ADHD, ODD, CD, and Autism Spectrum Disorder (50–52). However, since our study indicated that the prescription of atypical antipsychotics was more related to the overall severity of psychiatric symptom presentations, not specific categorical disruptive mood or behavior disorders, the effectiveness of prescribing antipsychotics in this fashion requires further inspection as well.

These findings lead to a speculation that the providers at the tertiary outpatient child psychiatry clinic, with experience in treating very sick patients, have already relied on the overall clinical assessment/impression of the degree of severity of collective psychopathologies as well as functional impairments, rather than pursued amelioration of symptom items based on categorical diagnosis (53). These results show that a dimensional approach to pediatric psychopathologies with latent individual analysis may provide insight into the current clinical practice in this particular real-life setting. Also, since the main decision in routine clinical practice is not generally whether to treat or not, but to match the intensity of the symptom presentation with the level of intervention (54), it may be natural that the intervention strategies correspond to overall dimensional severity of cluster of psychiatric symptoms than individual and/or comorbid diagnoses.

Lastly, it is critical to note that the youths in the severe/critical group experienced more frequent medication changes such as addition of new medications, increase of the dosages of existing medications, and changes to existing medications. This may reflect the real-world struggle of the providers who have to provide treatment for youths with severe level of psychopathologies, facing lack of clear guideline/algorithm or even evidence-based treatment options. Better characterization of the youths with severe psychopathologies by such methods applied in this study, as dimensional psychopathologies instead of categorical diagnoses, and latent profile analyses rather than variable analyses hopefully would guide the future direction of the clinical studies and treatment options.

There are a few caveats to offer. We only examined how the severity of indicators affected decision making for the current prescription and could not provide medication response such as the effectiveness and side effects. As of now we continue to do follow-up visits of these participants at 3, 6, 9, and 12 months, hoping to provide further insight into this question. The sample size in some disorders with low prevalence (i.e., CD/ODD) was not statistically valid in the analysis to identify the medication prediction, which might affect the statistical power. Likewise, it may not have been possible to detect the potential relationship between disorders with low prevalence and class membership. However, there are sufficient data in LPA and statistical analysis for identifying the differences between group separated by the LPA. Lastly, the trans-diagnostic indicators used in this study consisted mainly of assessing the severity of externalizing symptoms. This limits the generalization of our findings into the whole spectrum of childhood psychiatric symptoms by excluding the interpretations of internalizing disorders (i.e., depressive disorders, anxiety disorders, obsessive-compulsive and related disorders, trauma and stress-related disorders, and dissociative disorders) and relevant treatment intervention (i.e., SSRI, Anti-anxiety, Non-SSRI Antidepressant). However, this limitation is mitigated by the additional LPA analyses and their similar result to the main analysis, including internalizing psychopathologies (Tables S11–S13, Figures S2–S4). In future work it will be helpful to integrate the internalizing symptoms such as rumination (55) into our multidimensional approach, which may provide insight into common childhood pathophysiological mechanisms.

Despite some limitations, our results provide the empirical evidence for the utility of dimensional approach to understand childhood psychiatric disorders and treatment intervention. Externalizing psychopathologies (pediatric irritability, callous-unemotional trait, and aggression) seem to differentiate youths referred for psychiatric assessment and treatment at a tertiary child and adolescent psychiatric clinic as severe (high scores in all of these pathologies) and moderate (lower scores in all of these pathologies). Medication choices (antipsychotics, frequency of medication changes, and adding of psychiatric medications) by the providers are better explained by these clusters. We suggest these trans-diagnostic factors/psychopathologies to be taken into clinical practice and future research of child and adolescent psychiatry, in addition to the traditional categorical diagnoses.

## Data Availability Statement

The raw de-identified data supporting the conclusions of this article may be available upon request by the authors, without undue reservation.

## Ethics Statement

The studies involving human participants were reviewed and approved by University of Nebraska Medical Center (IRB Number 586-16). Written informed consent to participate in this study was provided by the participants' legal guardian/next of kin.

## Author Contributions

BS-V, RB, and SH conceived and planned the experiments. WG, AB, and AL carried out the experiments. RE and KC contributed to sample preparation. J-WS, RB, KP, and SH contributed to the interpretation of the results. SH took the lead in writing the manuscript. All authors provided critical feedback and helped shape the research, analysis and manuscript, contributed to the article, and approved the submitted version.

## Funding

This work was supported by the National Institute of Mental Health (U01MH120155) and IDeA CTR grant (1U54GM115458).

## Conflict of Interest

The authors declare that the research was conducted in the absence of any commercial or financial relationships that could be construed as a potential conflict of interest.

## Publisher's Note

All claims expressed in this article are solely those of the authors and do not necessarily represent those of their affiliated organizations, or those of the publisher, the editors and the reviewers. Any product that may be evaluated in this article, or claim that may be made by its manufacturer, is not guaranteed or endorsed by the publisher.

## Abbreviations

ADHD: Attention-Deficit/Hyperactivity Disorder
AIC: Akaike Information Criterion
ARI: Affective Reactivity Index
BIC: Bayesian Information Criterion
BLRT: Bootstrap Likelihood Ratio Test
CBCL: Child Behavior Checklist
CD: Conduct Disorder
CU: callous-unemotional trait
DBDs: disruptive mood and behavior disorders
ICL: Integrated Complete Likelihood
ICU: Inventory of Callous Unemotional Traits
LPA: latent profile analysis
ODD: Oppositional Defiant Disorder
PAQ: Proactive Aggression Questionnaire
RAQ: Reactive Aggression Questionnaire
SD: Standard Deviation
SSRI: serotonin selective reuptake inhibitor.
