# Supplementary material for: Research Audit on Clinical Utility of Dimensional Disruptive Mood and Behavior Psychopathologies in Child and Adolescent Psychiatry Practice
Source: Front Psychiatry. 2022 Apr 6;13:742148. doi: 10.3389/fpsyt.2022.742148 (PMC9020472; doi:10.3389/fpsyt.2022.742148)
Supplement: Supplementary file 1 [file Data_Sheet_1.docx]

Supplementary Material

# Inclusion criteria for study participants

a) Children aged between 5 and 18 years old; b) Children coming to the clinics for assessment/treatment for their emotional/behavioral problems; c) Children who can perform a neuropsychological battery; and d) Children who don’t have serious medical problems (such as brain tumor, cancer, or genetic disorders)

# Symptom profile measurements

## Aggression

The Reactive-Proactive Aggression Questionnaire (RAQ and PAQ, respectively) was administered as self- and parent-report to assess two types of aggressive behavior [1]. Comprised of 12 proactive aggression and 11 reactive aggression subscale items, youths and their caregivers rated aggressive behavior using a 3-point Likert scale (1 “Never” to 3 “Often”). The mean score for both subscales was computed with higher scores indicating more aggressive behavior. Evidence of construct validity and reliability has been demonstrated in previous publications [2; 3] and internal consistency of subscales for both parent and child report falls in the adequate to high range for this measure [4].

## Irritability

Both the participant and caregiver completed the Affective Reactivity Index (ARI), a 7-item inventory designed to assess irritability and emotion dysregulation [5]. Using a 3-point Likert scale ranging from 0 “Not true” to 2 “Certainly true,” parent- and self-report forms were used to rate the child’s irritable behavior (6 items; e.g., easily annoyed by others, often lose temper) and impairment (1 item; i.e., irritability causes problems) over the past 6 months. The first 6 items were summed to create a total score (range 0-12). Previous research has documented evidence of construct and discriminant validity, as well as excellent internal consistency regardless of reporting source [5; 6].

## Callous-Unemotional traits

The Inventory of Callous Unemotional Traits (ICU), a 24-item measure, was completed by both caregiver and youth [7]. The ICU captures three dimensions, including Callousness (11 items), Uncaring (8 items), and Unemotional (5 items). Responses were coded on a 4-point Likert scale, ranging from 0 “Not at all true” to 3 “Definitely true”. Support for construct validity and reliability has been documented in previous publications [8; 9].

## Behavioral and emotional problem measurements

The Child Behavior Checklist for Ages 6–18 (CBCL/6-18) [10] was administered to caregivers at baseline, and 3-month follow-up visits. The CBCL is composed of 7 items regarding a child’s academics, social life, and activities (e.g., “Does your child take part in sports?”) and 113 problem items, rated as 0 “Not true”, 1 “Somewhat or sometimes true”, or 2 “Very true or often true. Mean test-retest reliabilities have been reported to range from 0.95 to 1.00, and internal consistency has ranged from 0.78 to 0.97 [11]. The following CBCL subscales were included in the present study: 1) overall CBCL/6–18 summary scales including Total Problems and its Total Internalizing and Total Externalizing scales, 2) the competence scale including Total Competence and the Activities, Social, and School scales, 3) the 2007 scale including Sluggish Cognitive Tempo, Stress problems, and Obsessive Compulsive Problems, 4) the syndrome scales including Anxious/Depressed, Withdrawn/Depressed, Somatic Complaints, Social Problems, Thought Problems, Attention Problems, Rule Breaking Behavior, and Aggressive Behavior, and e) the CBCL DSM-oriented scales including Affective Problems, Anxiety Problems, Somatic Problems, ADHD Problems, Oppositional Defiant Problems, and Conduct Problems.

# Symptom profile changes in follow-up

Youths in both groups showed significant changes compared to baseline over time in almost all symptom profile scales. In other words, after three months, the scores of almost all symptom profiles except for parent-reported PAQ were significantly reduced (self-reported ARI, F= 14.54, p< .000; parent-reported ARI, F= 9.82, p= .003; self-reported ICU, F= 5.84, p= .018; parent-reported ICU, F= 14.73, p= .000; self-reported RAQ, F= 14.43, p< .000; parent-reported RAQ, F= 17.98, p< .000; self-reported PAQ, F= 4.26, p= .043). Also, significant interaction effects were shown in self-reported ARI (F= 13.15, p= .001), self-reported ICU (F= 5.72, p= .019), self-reported RAQ (F= 12.40, p= .001), and parent-reported RAQ (F= 14.13, p< .000) by times and groups; see Table 5. The severe/critical group showed a greater decrease in the self-reported ARI, self-reported ICU, self-reported RAQ, and parent-reported RAQ in the 3-month follow-up compared to the moderate group. However, even after significant reduction of all the symptoms, the critical/severe group still showed higher scores in all symptom profiles except for self-reported ICU than moderate group (self-reported ARI, t= 4.01, p< .000; parent-reported ARI, t= 4.68, p< .000; self-reported ICU, t= 1.22, p= .22; parent-reported ICU, t= 2.86, p< .01; self-reported RAQ, t= 4.74, p< .000; parent-reported RAQ, t= 4.12, p< .000; self-reported PAQ, t= 4.27, p< .000; parent-reported PAQ, t= 4.95, p< .000) and clinically significant levels of the symptoms: see Table S2 & S3.

# Identification and description of the latent classes derived from symptom profile

Table S1 shows the LPA fit indices is identified by increasing the number of latent classes sequentially to determine the optimal latent model. Because of the lowest AIC value and relatively higher entropy, the AIC and entropy results indicated a 4-class solution; AIC = 2858.78 and entropy = 0.95. The BIC results indicate a two-class solution showing the lowest BIC value (BIC = 3133.44). A bootstrap validation procedure with 999 successful replications confirmed that the two-class solution presented a better fit than the four-class solution (log likelihood = − 1341.43, p< .001). Of all of these fit statistics, the BIC and BLRT has been identified as performing the most reliably [17]. Therefore, the two-class solution was selected, by considering the ICL value, the posterior probability and the proportions of members in each class.

# Table S1. Criteria for model fit by different number of classes

| **Number of Classes** | **Model fit Criteria** | | | | |
| --- | --- | --- | --- | --- | --- |
|  | **AIC** | **BIC** | **ICL** | **BLRT (p value)** | **Entropy** |
| **1** | 3024.13 | 3158.89 | -3158.89 |  |  |
| **2** | 2860.87 | 3133.44 | -3146.38 | 233.67 (0.009) | 0.91 |
| **3** | 2861.47 | 3271.85 | -3295.51 | 130.43 (0.029) | 0.86 |
| **4** | 2858.78 | 3406.99 | -3415.40 | 72.96 (0.86) | 0.95 |
| **5** | 2876.10 | 3562.12 | -3567.40 | 83.40 (0.45) | 0.96 |

Abbreviations: AIC, Akaike information criterion; BIC, Bayesian information criteria; BLRT, Bootstrapped likelihood ratio test; ICL, Integrated Complete Likelihood.

# Table S2. Descriptive statistics on the indicator variables in each group (N=158)

| **Variables** | **The moderate group** | | | | **The severe/critical group** | | | |
| --- | --- | --- | --- | --- | --- | --- | --- | --- |
|  | **Initial (N=81)** | **Initial**  **(N=41)** | **Second (N=41)** | **t value** | **Initial (N=77)** | **Initial (N=33)** | **Second (N=33)** | **t value** |
| **ARIS** | 3.49 (2.71) | 2.93  (2.49) | 2.88 (2.54) | .143 | 6.82 (2.83) | 7.15  (2.65) | 5.21 (2.42) | 4.86*** |
| **ARIP** | 3.48 (2.85) | 3.26  (2.86) | 2.64 (2.56) | 1.71 | 7.01 (2.88) | 7.10  (2.77) | 5.67 (3.01) | 2.44* |
| **ICUS** | 21.79 (7.63) | 21.05  (6.48) | 21.03 (8.03) | .19 | 27.48 (8.63) | 27.30  (8.18) | 23.38 (8.49) | 3.09** |
| **ICUP** | 23.83 (11.45) | 24.01  (12.03) | 21.26 (10.75) | 2.40* | 32.97 (10.38) | 32.70  (11.53) | 28.42 (10.60) | 2.93** |
| **RAQS** | 6.90 (3.17) | 6.22  (2.85) | 6.10 (3.25) | .25 | 13.14 (4.15) | 13.30  (4.02) | 10.09 (3.99) | 4.24*** |
| **RAQP** | 7.73 (4.11) | 7.53  (4.03) | 7.31 (4.25) | .41 | 15.29 (3.77) | 15.57  (3.48) | 11.85 (5.24) | 4.51*** |
| **PAQS** | .60 (.77) | .46  (0.78) | .44 (.92) | .16 | 4.32 (3.46) | 3.82  (2.21) | 2.67 (3.19) | 1.89 |
| **PAQP** | 1.10 (1.20) | 1.05  (1.19) | .71 (1.11) | 2.26* | 6.41 (4.07) | 5.50  (4.25) | 3.71 (3.68) | 2.50* |

*P < 0.05, **P < 0.01, ***P < 0.001.

Paired sample t-test was conducted between initial (N=41, 33 in each group) and second (N=41, 33 in each group) reports.

Abbreviations: ARIS, Affective Reactivity Index self-report; ARIP, Affective Reactivity Index parent-report; ICUS, Inventory of Callous Unemotional Traits self-report; ICUP, Inventory of Callous Unemotional Traits parent-report; PAQS Proactive Aggression

The variables including ICU self-report and ICU parent-report are analyzed using two sample t test. The other variables are non-normal distributed and the Mann-Whitney U test was used to compare between groups for the variables.

# Table S3. Time (initial vs. second report) by groups (the moderate group, N=41 vs. The severe/critical group, N=33) repeated measures of ANOVA results.

| **Variables** | **Interaction effect of time and group** | | **Main effect of time** | | **Main effect of group** | |
| --- | --- | --- | --- | --- | --- | --- |
|  | ***F* value** | ***p* value** | ***F* value** | ***p* value** | ***F* value** | ***p* value** |
| **ARIS** | 13.15 | .001 | 14.54 | .000 | 38.26 | .000*** |
| **ARIP** | 1.55 | .218 | 9.82 | .003 | 34.81 | .000*** |
| **ICUS** | 5.72 | .019 | 5.84 | .018 | 6.89 | .011* |
| **ICUP** | .70 | .405 | 14.73 | .000 | 9.77 | .003** |
| **RAQS** | 12.40 | .001 | 14.43 | .000 | 63.71 | .000*** |
| **RAQP** | 14.13 | .000 | 17.98 | .000 | 47.99 | .000*** |
| **PAQS** | 3.915 | .052 | 4.26 | .043 | 62.74 | .000*** |
| **PAQP** | .32 | .573 | 1.38 | .245 | 52.43 | .000*** |

*P < 0.05, **P < 0.01, ***P < 0.001.

Abbreviations: ARIS, Affective Reactivity Index self-report; ARIP, Affective Reactivity Index parent-report; ICUS, Inventory of Callous Unemotional Traits self-report; ICUP, Inventory of Callous Unemotional Traits parent-report; PAQS Proactive Aggression Questionnaire self-report; RAQS Reactive Aggression Questionnaire self-report; PAQP Proactive Aggression Questionnaire parent-report; RAQP Reactive Aggression Questionnaire parent-report.

# Table S4. Correlation analysis between symptoms in all participants

|  | **ARI** | **ICU** | **RAQ** |
| --- | --- | --- | --- |
| **ICU** | .386** |  |  |
| **RAQ** | .785** | .448** |  |
| **PAQ** | .589** | .478** | .752** |

*P < 0.05, **P < 0.01, ***P < 0.001.

Abbreviations: ARI, Affective Reactivity Index; ICU, Inventory of Callous Unemotional Traits; RAQ Reactive Aggression Questionnaire; PAQ Proactive Aggression Questionnaire.

# Table S5. Correlation analysis between symptoms in the moderate group

|  | **ARI** | **ICU** | **RAQ** |
| --- | --- | --- | --- |
| **ICU** | .237* |  |  |
| **RAQ** | .631** | .198 |  |
| **PAQ** | .433** | .351** | .569** |

*P < 0.05, **P < 0.01, ***P < 0.001.

Abbreviations: ARI, Affective Reactivity Index; ICU, Inventory of Callous Unemotional Traits; RAQ Reactive Aggression Questionnaire; PAQ Proactive Aggression Questionnaire.

# Table S6. Correlation analysis between symptoms in the severe/critical group

|  | **ARI** | **ICU** | **RAQ** |
| --- | --- | --- | --- |
| **ICU** | .103 |  |  |
| **RAQ** | .628** | .211 |  |
| **PAQ** | .259* | .288* | .456** |

*P < 0.05, **P < 0.01, ***P < 0.001.

Abbreviations: ARI, Affective Reactivity Index; ICU, Inventory of Callous Unemotional Traits; RAQ Reactive Aggression Questionnaire; PAQ Proactive Aggression Questionnaire.

# Table S7. Comparison of correlations between the moderate and severe/critical groups.

|  | **The moderate group (N=81)** | **The severe/critical group (N=77)** | **Value (Z**) |
| --- | --- | --- | --- |
| **ARI-ICU^a^** | 0.242 | 0.103 | .852 |
| **ARI-RAQ^a^** | 0.743 | 0.738 | .031 |
| **ARI-PAQ^a^** | 0.464 | 0.265 | 1.224 |
| **ICU-RAQ^a^** | 0.201 | 0.214 | -.084 |
| **ICU-PAQ^a^** | 0.367 | 0.296 | .433 |
| **RAQ-PAQ^a^** | 0.646 | 0.492 | .948 |

The Fisher’s Z test was used to compare between groups for the variables.

aCorrelation coefficient values were transformed into z scores.

*P < 0.05, **P < 0.01, ***P < 0.001.

# Table S8. Differences between two dependent correlations in all participants

|  | **ARI-ICU** | **ARI-RAQ** | **ARI-PAQ** | **ICU-PAQ** | **ICU-RAQ** |
| --- | --- | --- | --- | --- | --- |
| **ARI-RAQ** | -6.88*** |  |  |  |  |
| **ARI-PAQ** | -3.00** | 5.31*** |  |  |  |
| **ICU-PAQ** | -1.44 | 4.52*** | 1.58 |  |  |
| **ICU-RAQ** | -1.31 | 5.71*** | 1.62 | 0.61 |  |
| **PAQ-RAQ** | -4.76*** | 0.85 | -4.52*** | -4.72*** | -5.29*** |

The Steiger’s Z test was used to compare between groups for the variables.

The values represent test Statistic z values.

*P < 0.05, **P < 0.01, ***P < 0.001.

# Table S9. Differences between two dependent correlations in the moderate groups.

|  | **ARI-ICU** | **ARI-RAQ** | **ARI-PAQ** | **ICU-PAQ** | **ICU-RAQ** |
| --- | --- | --- | --- | --- | --- |
| ARI-RAQ | -3.35*** |  |  |  |  |
| ARI-PAQ | -1.66 | 2.37* |  |  |  |
| ICU-PAQ | -1.01 | 2.24* | 0.66 |  |  |
| ICU-RAQ | 0.41 | 3.73*** | 1.61 | 1.54 |  |
| PAQ-RAQ | -2.45* | 0.70 | -1.68 | -1.84 | -3.324*** |

The Steiger’s Z test was used to compare between groups for the variables.

The values represent test Statistic z values.

*P < 0.05, **P < 0.01, ***P < 0.001.

# Table S10. Differences between two dependent correlations in the severe/critical groups.

|  | **ARI-ICU** | **ARI-RAQ** | **ARI-PAQ** | **ICU-PAQ** | **ICU-RAQ** |
| --- | --- | --- | --- | --- | --- |
| **ARI-RAQ** | -4.26*** |  |  |  |  |
| **ARI-PAQ** | -1.15 | 3.68*** |  |  |  |
| **ICU-PAQ** | -1.35 | 2.58** | -0.20 |  |  |
| **ICU-RAQ** | -1.10 | 3.25** | 0.31 | 0.66 |  |
| **PAQ-RAQ** | -2.34* | 1.59 | -2.16* | -1.29 | -1.94 |

The Steiger’s Z test was used to compare between groups for the variables.

The values represent test Statistic z values.

*P < 0.05, **P < 0.01, ***P < 0.001.

# Table S11. LPA using nine indicators (self & parent-report ARI, self & parent-report ICU, self & parent-report reactive aggression, self & parent-report proactive aggression, and internalizing problems)

| **Number of Classes** | **Model fit Criteria** | | | | |
| --- | --- | --- | --- | --- | --- |
|  | **AIC** | **BIC** | **ICL** | **BLRT (p value)** | **Entropy** |
| 1 | 3485.00 | 3650.38 | -3650.38 |  |  |
| *2* | *3290.04* | *3623.87* | *-3631.33* | *308.61(0.00)* | *0.91* |
| 3 | 3304.12 | 3806.39 | -3814.87 | 100.04(0.71) | 0.94 |
| 4 | 3318.36 | 3989.07 | -3993.70 | 109.84(0.39) | 0.97 |
| 5 | 3274.03 | 4413.18 | -4117.21 | 117.00(0.10) | 0.98 |

# Table S12. LPA using 11 indicators (self & parent-report ARI, self & parent-report ICU, self & parent-report reactive aggression, self & parent-report proactive aggression, withdrawn-depressed score, depressive problems, and anxiety problems)

| **Number of Classes** | **Model fit Criteria** | | | | |
| --- | --- | --- | --- | --- | --- |
|  | **AIC** | **BIC** | **ICL** | **BLRT (p value)** | **Entropy** |
| 1 | 4184.83 | 4420.65 | -4420.65 |  |  |
| *2* | *4043.81* | *4518.52* | *-4519.90* | *282.83(0.01)* | *0.98* |
| 3 | 4042.91 | 4756.49 | -4761.60 | 101.42(0.96) | 0.96 |
| *4* | *3953.13* | *4905.60* | *-4910.19* | *219.70(0.02)* | *0.97* |
| 5 | 4106.67 | 5298.01 | -5300.59 | 83.73(0.90) | 0.98 |

# Table S13. LPA using 12 indicators (self & parent-report ARI, self & parent-report ICU, self & parent-report reactive aggression, self & parent-report proactive aggression, anxious-depressed score, withdrawn-depressed score, depressive problems, and anxiety problems)

| **Number of Classes** | **Model fit Criteria** | | | | |
| --- | --- | --- | --- | --- | --- |
|  | **AIC** | **BIC** | **ICL** | **BLRT (p value)** | **Entropy** |
| 1 | 4325.21 | 4600.85 | -4600.85 |  |  |
| 2 | 4130.69 | 4685.02 | -4687.39 | 370.06(0.01) | 0.96 |
| 3 | 4125.49 | 4958.51 | -4962.73 | 171.38(0.30) | 0.97 |
| 4 | 4184.41 | 5296.14 | -5297.84 | 144.87(0.65) | 0.98 |


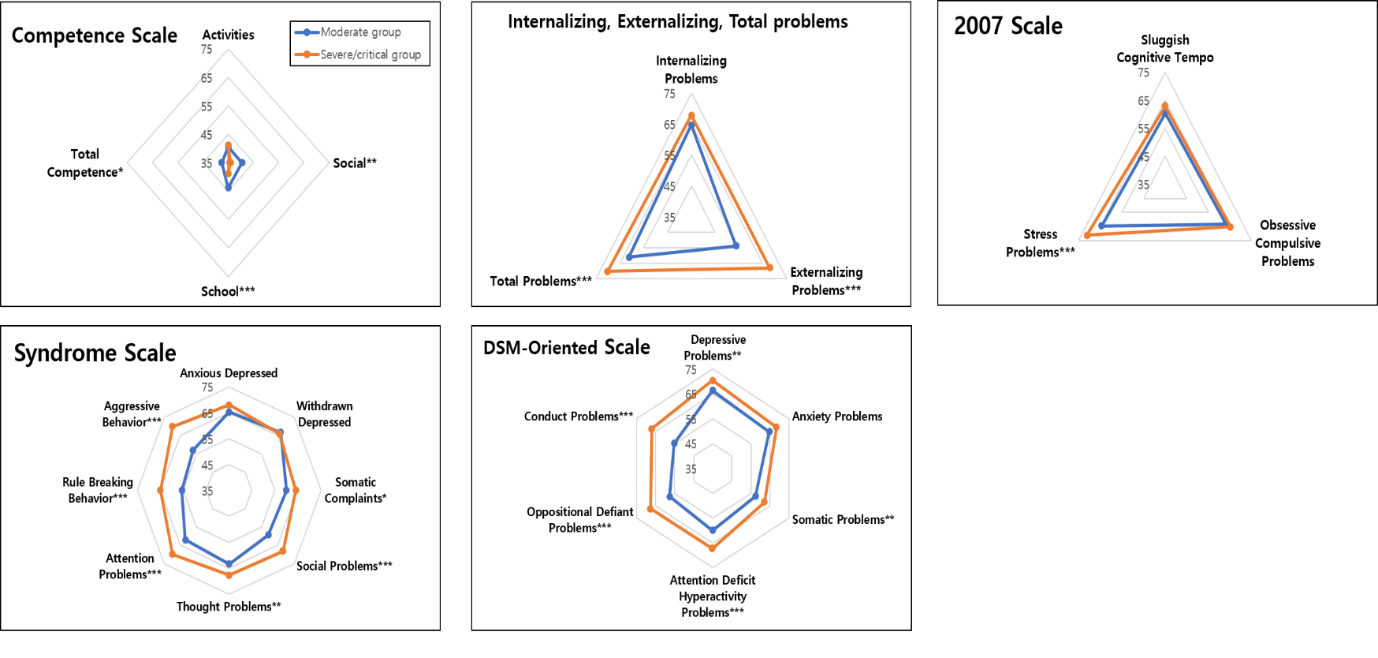


# Figure S1. Differences of the scores at baseline in CBCL sub-scales between two Latent Classes (N=158)

Abbreviations: CBCL, Child Behavior Checklist.

*P < 0.05, **P < 0.01, ***P < 0.001.

The variables including externalizing problems and depressive problems are analyzed using two sample t test. The other variables are non-normal distributed and the Mann-Whitney U test was used to compare between groups for the variables.

# Figure S2. LPA using nine indicators (self & parent-report ARI, self & parent-report ICU, self & parent-report reactive aggression, self & parent-report proactive aggression, and internalizing problems)

# Figure S3. LPA using 11 indicators (self & parent-report ARI, self & parent-report ICU, self & parent-report reactive aggression, self & parent-report proactive aggression, withdrawn-depressed score, depressive problems, and anxiety problems)

# Figure S4. LPA using 12 indicators (self & parent-report ARI, self & parent-report ICU, self & parent-report reactive aggression, self & parent-report proactive aggression, anxious-depressed score, withdrawn-depressed score, depressive problems, and anxiety problems)

**References**

[1] K.A. Dodge, and J.D. Coie, Social-information-processing factors in reactive and proactive aggression in children's peer groups. Journal of personality and social psychology 53 (1987) 1146.

[2] K.A. Dodge, J.E. Lochman, J.D. Harnish, J.E. Bates, and G.S. Pettit, Reactive and proactive aggression in school children and psychiatrically impaired chronically assaultive youth. Journal of abnormal psychology 106 (1997) 37.

[3] D.A. Waschbusch, and M.T. Willoughby, Criterion validity and the utility of reactive and proactive aggression: Comparisons to attention deficit hyperactivity disorder, oppositional defiant disorder, conduct disorder, and other measures of functioning. Journal of Clinical Child Psychology 27 (1998) 396-405.

[4] P.J. Fite, L. Stoppelbein, and L. Greening, Proactive and reactive aggression in a child psychiatric inpatient population. Journal of Clinical Child & Adolescent Psychology 38 (2009) 199-205.

[5] A. Stringaris, R. Goodman, S. Ferdinando, V. Razdan, E. Muhrer, E. Leibenluft, and M.A. Brotman, The Affective Reactivity Index: a concise irritability scale for clinical and research settings. Journal of Child Psychology and Psychiatry 53 (2012) 1109-1117.

[6] M.A. Mulraney, G.A. Melvin, and B.J. Tonge, Psychometric properties of the affective reactivity index in Australian adults and adolescents. Psychological assessment 26 (2014) 148.

[7] P. Frick, The Inventory of Callous-Unemotional traits: Unpublished rating scale; 2004, 2004.

[8] E.R. Kimonis, P.J. Frick, J.L. Skeem, M.A. Marsee, K. Cruise, L.C. Munoz, K.J. Aucoin, and A.S. Morris, Assessing callous–unemotional traits in adolescent offenders: Validation of the Inventory of Callous–Unemotional Traits. International journal of law and psychiatry 31 (2008) 241-252.

[9] C.A. Essau, S. Sasagawa, and P.J. Frick, Callous-unemotional traits in a community sample of adolescents. Assessment 13 (2006) 454-469.

[10] T.M. Achenbach, L. Dumenci, and L.A. Rescorla, Ratings of relations between DSM-IV diagnostic categories and items of the CBCL/6-18, TRF, and YSR. Burlington, VT: University of Vermont (2001) 1-9.

[11] T.M. Achenbach, and C. Edelbrock, Child behavior checklist. Burlington (Vt) 7 (1991) 371-392.

[12] L. Muthén, and B. Muthén, MPlus for windows 7.31. Los Angeles: Muthén & Muthén (2015).

[13] H. Akaike, Factor analysis and AIC, Selected papers of hirotugu akaike, Springer, 1987, pp. 371-386.

[14] G. Schwarz, Estimating the dimension of a model. The annals of statistics (1978) 461-464.

[15] C. Biernacki, G. Celeux, and G. Govaert, Assessing a mixture model for clustering with the integrated completed likelihood. IEEE transactions on pattern analysis and machine intelligence 22 (2000) 719-725.

[16] G.J. McLachlan, On bootstrapping the likelihood ratio test statistic for the number of components in a normal mixture. Journal of the Royal Statistical Society: Series C (Applied Statistics) 36 (1987) 318-324.

[17] K.L. Nylund, T. Asparouhov, and B.O. Muthén, Deciding on the number of classes in latent class analysis and growth mixture modeling: A Monte Carlo simulation study. Structural equation modeling: A multidisciplinary Journal 14 (2007) 535-569.
